# Human Herpes Virus Type 6 and Febrile Convulsion

**Published:** 2015

**Authors:** Mohammad Mehdi HOUSHMANDI, Alireza MOAYEDI, Mohammad Bagher RAHMATI, Abdulmajid NAZEMI, Darioush FAKHRAI, Shahram ZARE

**Affiliations:** 1.Pediatrician, Clinical Research Development Center of Children Hospital, Hormozgan University of Medical Sciences, Bandar Abbas, Iran; 2 Department of Pediatrics Neurology, Pediatric Hospital, Hormozgan University of Medical Sciences, Bandar Abbas, Iran 3.; 3 Clinical Research Development Center of Children Hospital, Hormozgan University of Medical Sciences, Bandar Abbas, Iran; 4 Department of Pediatrics, Faculty of Medicine, Hormozgan University of Medical Sciences, Bandar Abbas, Iran; 5 General Practitioner, Clinical Research Development Center of Children Hospital, Hormozgan University of Medical Sciences, Bandar Abbas, Iran; 6 Department of Community Medicine, Hormozgan University of Medical Sciences, Bandar Abbas, Iran

**Keywords:** HHV-6, Children, Febrile convulsion

## Abstract

**Objective:**

Febrile Convulsion (FC) is occurred in 6 months to 5 yr old children. The aim of this study was to investigate the prevalence of HHV-6 infection in FC admitted patients of Bandar Abbas Children Hospital, southern Iran.

**Materials & Methods:**

In a cross-sectional study, 118 children aged 6-60 months who had FC were selected by a simple random method in 2010-11. Demographic data, clinical manifestation and two blood samples gathered to assess the human herpes virus type 6 (HHV6). Blood sample obtained at the time of admission and 10 days

after the first examination. ELISA was used to detect HHV-6 IgG. The subjects were studied in two groups with and without infection of HHV-6. Two groups were compared by t-test and X2.

**Results:**

Fifty-three subjects completed the study, including 30 boys (56.6 %) and 23 girls (43.4%). The HHV-6 infection was detected in 23 patients out of 53 studied subjects. The mean of age for the groups with and without HHV-6 infection was 19.7±9.7 and 20.4±10.2 months old, respectively. The most common clinical presentation in both groups was rhinorrhea, diarrhea, vomiting and lethargy without any significant difference between two groups. Five patients (21.7%) in HHV-6 group and 1 patient (3.3%) in HHV-6 negative group had postictal phase more than 15 minutes (P<0.05). Convulsion within 1 hour from beginning of fever was more frequent in HHV-6 infection group than the other group (P<0.01).

**Conclusion:**

There was not any difference in terms of age group, gender and clinical manifestation of infected and non-infected children with FC. Postictal phase and seizure during 1 hour after the fever were significantly different between two groups.

## Introduction

Most cases of Febrile Convulsions (FC) happen in children ages between 6 months to 5 yr old with a peak at 14-18 months old (1). Recently, researchers have focused on human herpes virus type 6 (HHV-6) as the etiology of 20-25% of the FC cases (2). Primary infection of HHV-6 has been associated with exanthema subitum known as Roseola infantum (6th Disease or three days fever) (3). Febrile illness, rash and convulsion are clinical symptom associated with HHV-6 infection. A supposed role of HHV-6 in central nervous system is under investigation. Other clinical presentations include fever without any focus, fever and convulsion, convulsion disease, meningitis, meningoencephalitis and demyelination disease (4). Primary HHV- 6 infection is correlated with 26-43% of first episode of convulsion in patients with febrile seizure (5), and 8-20% of patient FC as first presentation (6). The present study aimed to investigate the prevalence of HHV-6 infection in FC admitted patients of Bandar Abbas Children Hospital.

## Materials & Methods

A cross-sectional study was conducted among children aged 6-60 months old referred to Bandar Abbas Children Hospital, southern Iran from October 2010 –October 2011 due to their febrile convulsion. Including Criteria were 1) Occurrence of FC in patients between 6 months to 5 yr old; 2) Associated fever described as an axillary temperature of 37.4 or greater (7); 3) Convulsion criteria included focal or generalized, tonic (increased muscle tone) and clonic (rhythmic contraction and relaxation of muscles) seizure attack associated with fever (8). Excluding criteria were meningitis, encephalitis, acute electrolyte imbalance, serious illness like sepsis, previous history of convulsion, history of neonatal convulsion, family history of FC or convulsion, and a history of treatment with anticonvulsants. According to the including criteria, 118 patients were selected by a simple randomized method. The study was approved by the Ethics Committee of Hormozgan University of Medical Sciences and informed consent was taken from parents. A blood sample about two ml was obtained at the time of admission and 10 days after the first examination based on kit guideline to detect the HHV -6 infection. Sera were obtained within 24 h after blood collection by a 2500 rpm centrifuge during 10 min and stored in a freezer (-40 °C) for 3 months to do the HHV-6 test. HHV-6 IgG was detected by ELISA with Biotrin ELISA kit Ireland with sensitivity of 97.4% and specificity of 86% for HHV-6 IgG. Demographic data included age, sex, clinical presentations (fever, rash, neck rigidity, Kernig and Brudzinski, lethargy), body temperature, headache, interval between fever and convulsion occurrence, type of convulsion (simple or complex) gathered. White blood cell (WBC) count, erythrocyte sedimentation rate (ESR), C Reactive Protein (CRP), Na, blood culture were assessed and if the possible lumbar puncture of the patients were evaluated. CSF was analyzed for glucose, protein, WBC count and culture and blood sugar detected. Reported results were for the patient who had two blood samples including the admission time and 10 days afterwards. About 65 subjects did not complete the study because the parents did not agree to take the second blood sample or were far from the hospital. The mention children were not included in final analysis. Convulsion within 1 hour from beginning of fever and postictal phase duration was recorded. Patients were divided into two groups, with and without HHV-6 infection. Data were presented as mean± SD. The software of SPSS version 16 (Chicago, IL, USA) was used for data analysis. t-test, Mann Whitney, and X2 were used. P<0.05 was considered as a significant difference.

## Results

Fifty-three patients out of 118 studied subjects enrolled into the study, about 43% of the patients had HHV- 6 infection, including 11 boys and 12 girls (43.4%) and the mean of their age was 19.7±9.7 months old. Mean age in HHV-6 negative test, including 11 female and 29 male patients, was 20.4 ± 10.2 months. There was no significant difference between two groups for age and gender distribution. The most common clinical presentation in both group was rhinorrhea due to upper respiratory infection, diarrhea and vomiting as a result of gastroenteritis and lethargy due to fever without any focus, and there were not any significant different between two groups ( Table1). Mean and standard deviation of WBC in HHV-6 infected group was 9661± 4225 in cube millimeter and in non HHV-6 infection group 11541±6188 without any significant difference between two groups. There was no significant difference in laboratory data between two groups. In comparison between ESR of infected and non- infected children was 22.6±15.1 ml (mm) per hour vs.19.2±12.2, respectively. ESR in 43.5% patients with HHV-6 infection was more than 11 mm per h compared with patients without HHV-6, which was more than 11 mm per h. CRP was negative in 34.8% of patients in HHV-6 infection group and 56.7% of the other group. In both group infected and none infected with HHV- 6, there was polymorphonuclear cell dominancy in peripheral blood smear. Mean percent of neutrophil predominance for children with and without primary HHV-6 infection was 56.6% and 60%, respectively. Five patients (21.7%) in HHV-6 group had postictal phase more than 15 min and only 1 patient (3.3%) in HHV-6 negative group had postictal which was significant (P<0.05).

The incidence of convulsion within 1 h from beginning of fever was more frequent in HHV-6 infection group than the other group; 7 vs. 2 patients, respectively (P=0.02).

**Table 1:** Frequency of clinical symptom in HHV-6 infection positive and Negative patients

**Clinical Symptom**	**HHV -6 Infection**		***P*** **-value**
	**Positive n (%) **	**Negative (%)**	
Rash	1(4.3)	1(3.3)	0.49
Lethargy	16 (69.5)	10 (33.3)	0.13
Nasal Congestion	13 (56.5)	20 (66.6)	0.59
Diarrhea & Vomiting	10 (43.3)	14 (46.6)	0.89
Lower Respiratory Infection	8 (34.7)	11 (36.6)	0.71

## Discussion

Febrile seizures are not the result of central nervous system infection or any metabolic imbalance, and that occur in the absence of a history of prior afebrile seizures. It is often due to otitis media, roseola and HHV6 infection, Shigella, or similar infections (8). In our study, overall infection with HHV-6 was 43%. Herpes infections incidence was 3-18% and 26-44% (9-14).The considerable difference in the incidence of HHV-6 infection in children might be related to demographic characteristic of the studied group such as age of patients or different sensitivity and specificity of the particular assays. Obviously, infection prevalence is higher in children younger than 2 yr old, so the figures are significantly higher in this age group than another study (15). Hall et al. reported a greater proportion of febrile children between 12-15 months of age with primary HHV-6 infection who had febrile seizures than control group without HHV-6 infection; 8 out of 22 children vs. 17 out of 131 subjects (16). In this study, children with HHV-6 infection aged less than 24 months old were subjects (78.3%). Different methods for HHV assay such as PCR and indirect immunofluorescent assay could be another explanation for observed differences. There was not any gender difference for febrile seizures with HHV infection and sex distribution was equal in both group of with and without HHV-6 infection, which was in accordance to the results of Greek children (6), but in the United States, female subjects were more prone to HHV-6 infection (17). Changes in white blood cell (WBC) counts were similar to Feach et al. (18). Neutrophil cells were dominant to the other type of blood cell in HHV6 infection group, which is not investigated in the other studies and need to be surveyed. The highest rate of seizures was during one h after the febrile incidence and there were not any pleocytosis of CSF like the result of Teach et al. (18). The possible explanation could be that febrile seizures are not induced by direct viral attack to the brain; it is the result of vasculitis by viruses’ bodies or secretion of toxins after viral infection (18). Clinical manifestations were not different between two groups of with and without HHV-6 infection. Zerr et al. observed a higher rate of gastrointestinal signs in group with HHV-6 infection without any significant difference (19). In this study, lethargy due to fever without any focus was more prevalent in the group with HHV-6 infection but it was not significantly different. Postictal lethargy was considerably different in two groups, which was similar to the results of Suga et al. (15). ELISA was used for HHV-6 infection which might be included as a limitation in this study, but this kit had a lower cost in comparison to IF and PCR methods, the reliability and validity of assessment method was good enough for HHV-6, so it can be a relatively proper method for screening of febrile seizures patients in hospital as an alternative for assessment of acute HHV-6 infection and an effective treatment of disease, although it seems that PCR is the most recommended one for children. HHV infection does not need treatment, but it can be one of the FC‘s causes. Primary HHV-6 infection is frequently associated with febrile seizures in children and especially should be considered for first episode of febrile seizures (6, 15). Children who develop seizures in response to these common viruses may harbor genes that cause them to be susceptible (20). The virus often results in the development of a more severe form of convulsions, such as partial seizures, prolonged seizures, and repeated seizures. It might be a risk factor for subsequent development of epilepsy (15), so a longitudinal study for children with HHV-6 infection is recommended to investigate the effects of the infection in a long period.

**In conclusion**, the highest incidence (76.3%) of febrile seizure in children with HHV-6 infection was in the age group of <24 months old. Clinical manifestation and gender distribution were not different between two groups with and without HHV-6 infection. Postictal phase and seizure during 1 hour after the fever were significantly different between two groups
